# cGAS in nucleus: The link between immune response and DNA damage repair

**DOI:** 10.3389/fimmu.2022.1076784

**Published:** 2022-12-15

**Authors:** Jia-Xian Song, Deana Villagomes, Hongchang Zhao, Min Zhu

**Affiliations:** ^1^ Institute for Translation Medicine, The Affiliated Hospital of Qingdao University, College of Medicine, Qingdao University, Qingdao, China; ^2^ Department of Molecular and Cellular Biology, University of California Davis, One Shields Avenue, Davis, CA, United States; ^3^ Department of Microbiology and Molecular Genetics, University of California Davis, One Shields Avenue, Davis, CA, United States

**Keywords:** cGAS, DDR, innate immunity response, cancer therapy, post-translational modification

## Abstract

As the first barrier of host defense, innate immunity sets up the parclose to keep out external microbial or virus attacks. Depending on the type of pathogens, several cytoplasm pattern recognition receptors exist to sense the attacks from either foreign or host origins, triggering the immune response to battle with the infections. Among them, cGAS-STING is the major pathway that mainly responds to microbial DNA, DNA virus infections, or self-DNA, which mainly comes from genome instability by-product or released DNA from the mitochondria. cGAS was initially found functional in the cytoplasm, although intriguing evidence indicates that cGAS exists in the nucleus where it is involved in the DNA damage repair process. Because the close connection between DNA damage response and immune response and cGAS recognizes DNA in length-dependent but DNA sequence–independent manners, it is urgent to clear the function balance of cGAS in the nucleus versus cytoplasm and how it is shielded from recognizing the host origin DNA. Here, we outline the current conception of immune response and the regulation mechanism of cGAS in the nucleus. Furthermore, we will shed light on the potential mechanisms that are restricted to be taken away from self-DNA recognition, especially how post-translational modification regulates cGAS functions.

## Innate immunity response: The pivotal role of pattern recognition receptors

To resist the invasion of pathogens, innate immunity is gradually formed during the long-term evolution of organisms, which recognize exogenous pathogen-associated molecular patterns and endogenous damage-associated molecular patterns through pattern recognition receptors (PRRs). PRR mainly includes Toll-like receptors (TLRs), retinoic acid–inducible gene-I (RIG-I)–like receptors (RLRs), nucleotide oligomerization domain (NOD)–like receptors (NLRs), C-type lectin receptors (CLRs), and a series of intracellular nucleic acid sensors (1, 2).

As type I transmembrane glycoproteins, TLRs consist of an extracellular domain that recognizes pathogen components, a transmembrane domain that determines subcellular localization, and an intracellular signaling domain that is responsible for interacting with intracellular linker molecules. Ten TLRs (TLR1 to TLR10) have been identified in humans, mainly acting through two signal transduction pathways, the Myeloid differentiation primary response 88 (MyD88)-dependent pathway and the TIR-domain-containing adaptor protein inducing interferon-β (IFNβ) (TRIF)-dependent pathway, depending on the adaptor protein (3–5). Nucleic acid–sensitive TLRs (TLR3, TLR7, TLR8, TLR9, and TLR10) are located in the endosomal compartment (6), whereas other TLRs that recognize lipopeptides, lipopolysaccharide, and flagellin are located on the plasma membrane. Of the nucleic acid–sensitive TLRs, TLR3 recognizes and binds double-stranded RNA (dsRNA) with base specificity–independent electrostatic interactions (7), TLR7 (human TLR8) recognizes single-stranded RNA (ssRNA), and TLR9 recognizes unmethylated CpG islands (CpGs) containing ssDNA (8). All the above four nucleic acid receptors are expressed as homodimers on the surface of immune cells to recognize nucleic acids of microorganisms (9), of which TLR8 and TLR9 exist as dimers (10). Recognition of RNA and DNA by TLRs is restricted to the endosomal compartment of immune cells. TLR10 predominantly localized in endosomes is the least characterized TLR10 due to it being a pseudo-gene in mice. Two studies suggested TLR10 may suppress Myd88 signaling by binding dsRNA (11, 12).

RLR mainly detects exogenous cytoplasmic RNA of dsRNA viruses, and the dsRNA replication intermediates of ssRNA viruses (13–15). Among the RLR family, the receptors RIG-I, melanoma differentiation gene 5 (MDA5), and laboratory of genetics and physiology 2 (LGP2) are DExH/D box helicases (16). The DexD/H helicase domain of RLRs has ATPase and helicase activities, and the C-terminal domain recognizes viral RNA and undergoes conformational changes, whereas the N-terminal caspase activation and recruitment domain (CARD) is responsible for downstream signal transduction (17). Recognition of viral dsRNAs is mediated by RIG-I and MDA5, where relatively short (<1,000 bp) viral dsRNAs are recognized by RIG-I and long-chain dsRNAs (>2,000 bp) are recognized by MDA (18). LGP2 has no signal transduction function because it has no CARD domain (19), but it can regulate the signal transduction of RIG-I and MDA5 (20).

The NLR consists of a central nucleotide-binding domain for nucleic acid binding and oligomerization, a C-terminal LRR (leucine-rich repeat) domain that recognizes ligands and an N-terminal domain (21). NOD1 and NOD2 are the most typical NLR family PRRs, among which NOD1 recognizes diaminopimelic acid in Gram-negative bacteria, and NOD2 recognizes muramyl dipeptide in bacterial cell walls (22). NOD2 also recognizes intact viral ssRNA, which, in turn, activates interferon (IFN) production and anti-viral defense (23).

CLRs, which recognize not only pathogen moieties for host defense but also modified self-antigens, are a family of transmembrane PPRs (24). Dectin-1 and Dectin-2 are the most PRR-characterized CLRs, whereas Dectin-1 signaling controls nuclear factor κB (NF-κB) and Nuclear factor of activated T cells (NF-AT) activation, and Dectin-2 binds to the Immunoreceptor tyrosine-based activation motif (ITAM)-bearing molecule The γ subunit of the immunoglobulin Fc receptor (FcRγ) for signaling (25, 26).

## DNA sensors in cytosol

Many DNA sensors exist in the cytoplasm; although some of them can also respond to RNA, here, we talk about the group of sensors that can trigger the immune response under DNA attack, in which some of them are particularly in the immune response pathway; although some are classical DNA repair factors, these DNA repair factors exist in both nucleus and cytosol, and they execute different functions under certain conditions. These DNA sensors including DNA-dependent activator of IFN-regulatory factors (DAI), LRR (in flightless I) interacting protein-1 (LRRFIP1), DEAD (Asp-Glu-Ala-Asp) Box Polypeptide 41 (DDX41), absent in melanoma 2 (AIM2), p202, IFN-γ–inducible protein 16 (IFI16), DHX36 and DHX9, high-mobility group box (HMGB), Ku70, MRE11, cyclic GMP-AMP (cGAMP) synthase (cGAS) (27–41). DAI, also known as Z-DNA binding protein 1 and DLM-1, due to its ability to bind dsDNA, was identified as the first cytoplasmic DNA sensor after TLR9, activating NF-κB by binding TRAF family member-associated NF-kappa-B activator (TANK)-binding kinase 1 (TBK1) and the IFN regulatory factor 3 (IRF3) complex (28). However, whether DAI plays a relevant role in DNA sensing requires further experimental verification. LRRs are key motifs in the TLR domain responsible for recognizing PRRs (42), and the cytoplasmic localized LRRFIP1 was found to bind dsDNA and dsRNA, but not ssDNA and ssRNA (43). With several members of the DExD/H-box helicase superfamily identified as RNA sensors, DDX41 was also found to bind DNA and interact with STING through its DEADc (Asp-Glu-Ala-Asp) domain (44). AIM2, p202, and Interferon Gamma Inducible Protein 16 (IFI16) all are PYHIN family proteins, containing Pyrin and HIN domains (p202 only contains the DNA-binding HIN domain). AIM2 recognizes cytoplasmic dsDNA and forms an inflammasome that activates caspase-1 with Apoptosis-associated speck-like protein containing a CARD (ASC) (45). Nuclear-localized IFI16 mediates type I IFN responses by translocating to the cytoplasm depending on the DNA in the microenvironment (46). Both CpG-B–binding DHX9 and CpG-A–binding DHX36 are localized in the cytoplasm and directly bind to the Toll-interleukin receptor domain of MyD88 through their helicase-associated domain and Domain of unknown function (DUF) domains (47). This suggests that MyD88-dependent DNA sensors DHX9/DHX36 in the pDC cytosol have a broader role in virus sensing. The HMGB protein family is essential for maintaining the normal function of chromosomes and participates in various biological activities in the nucleus such as DNA replication, DNA transcription, and DNA repair (48, 49). It has a high affinity for DNA and RNA, thus promoting the activation of intracellular DNA receptors such as TLR and RIG-I in response to its nucleic acid ligands (50). Among them, HMGB2 mainly binds to DNA, whereas HMGB1 and HMGB3 can simultaneously bind to DNA and RNA (50). Ku70 is an evolutionarily conserved protein that forms a complex with DNA-PKcs and Ku80 during the Non-homologous end joining (NHEJ) DNA repair process (51). However, Ku acts as a cytosolic DNA sensor that ultimately induces type III but not type I IFN responses mediated by the IRF1 and IRF7 signaling pathways (52). Meiotic recombination homolog A (MRE11) specifically binds to dsDNA and promotes its repair during cellular damage but does not recognize DNA introduced by viruses (53). All of the above cytosolic pathogens recognition binders are either cell type–dependent or function in a sequence-specific manners. In 2013, the Sun et al. discovered that cGAS can recognize the cytosol DNA, and, since then, many fascinating studies are coming out to dissect its molecular mechanisms (54). To date, cGAS may be the only “real” DNA sensor (55), and it can directly bind to dsDNA and coordinate with the stimulator of IFN genes (STING) to activate TBK1 and IRF3 and then initiate the production of type I IFN or triggering the inflammation reaction through the NF-κB pathway. Among these identified DNA sensors that can induce inflammatory responses, Ku70 recognizes DNA to induce type III IFN production (37, 56), AIM2 inflammasome produces IL-1β and IL-18, whereas others induce type I IFNs through the TBK1–IRF3 signaling axis. A variety of DNA sensors induce the production of immune active factors, which may all initiate downstream signaling pathways in a STING-dependent manner. However, their molecular mechanisms of STING activation are still unclear (57–59). At present, it is relatively clear that cGAS catalyzes the synthesis of the second messenger cGAMP after recognizing dsDNA, which further binds and activates STING and then activates the transcription factors IRF3 and NF-κB to induce the production of type I IFN. Beyond the canonical role of cGAS in immune response in the cytosol, growing evidence indicates the critical roles of cGAS in the nucleus that is involved in the DNA damage repair process (60–63), which provides another layer of the cross-talk between immune response and DNA damage repair.

## cGAS-STING pathway: Central player in immune response

Wu et al. found that cGAMP as a second message can activate type I IFN when cells are transfected with DNA or infected with DNA virus. cGAS was identified as an enzyme that synthesizes cGAMP by the same group. Meanwhile, cGAS-STING–initiated immune response entered people’s field of vision (64). cGAS is a 520–amino acid–long nucleotidyl transferase comprising a highly basic unstructured N-terminus of 160 amino acids and a C-terminus containing a highly conserved NTase domain and a male abnormal 21 (Mab21) domain (65). The NTase domain is a common domain of the NTase superfamily, in which conserved amino acid residues such as D227, D319, E225, G212, and S213 are the key to the enzymatic function of cGAS, catalyzing the transfer of nucleoside phosphate to hydroxyl acceptor (41, 66). cGAS utilizes its positively charged surface and the zinc-ribbon domain of Mab21 to insert into the minor groove of DNA and interact with the sugar-phosphate backbone (67, 68), indicating that the interaction between cGAS and DNA is sequence-independent. To achieve the stable catalytic activity, cGAS needs to assemble into a dimer (69, 70). Whereas, two DNA-binding sites (A and B) on its catalytic domain cover about 16–18 bp of DNA, sandwiching the DNA strand between the dimer. Human cGAS dimers have a C site that cooperates with the N-terminal domain to promote liquid-phase condensation and are critical for cGAS activation (71). The dsDNA-binding ability of cGAS uses ATP and GTP as raw materials to synthesize the second messenger cGAMP, which, in turn, binds and activates the STING located in the endoplasmic reticulum. ER-localized STING is activated upon binding to cGAMP and translocated to the Golgi apparatus. At this point, the cGAS-STING complex activates kinase 1 (TBK1) and IκB kinase, which activate IRF3 and NF-κB, respectively, inducing the production of type I IFNs and other cytokines (72–75).

cGAS recognizes dsDNA and activates the immune response to produce type I IFN in a STING-dependent manner. Therefore, the cGAS-STING pathway is crucial for the identification and clearance of DNA-causing microorganisms. A variety of DNA viruses, including Adenovirus, herpes simplex virus (HSV), vaccinia virus, hepatitis B virus, pseudorabies virus, and cytomegalovirus, induce the production of type I IFNs through the cGAS-STING signaling pathway (76). Like all other pathogen-recognizing receptors, cGAS-STING–mediated innate immune response activation is affected by different factors, such as cell type, the pathogen intermediates, its redundant stimulators, or the cross-talk immune pathways (77, 78); this makes cGAS-STING–mediated immune response more elegant and complex. Retroviruses (such as HIV) enter macrophages and dendritic cells (DCs), usually without eliciting a strong innate immune response due to being masked by viral or host factors; under permission conditions, cGAS produces cGAMP through taking advantage of the HIV reverse transcription and can rekindle the immune response (79, 80); cGAS-generated cGAMP can also be packaged into the virus particles and extracellular vesicles, which can be delivered to the DCs and then can activate the innate immune response (81). The following study provides evidence that the transmembrane transporter of cGAMP, the anion channel LRRC8/VRAC, can transport cGAMP from HSV-1 infected cells to non-infected cells, thereby exerting anti-viral effects (82). Cell-to-cell contact and transmission is another strategy for the innate immune cells to execute the cGAS-dependent anti-virus function. HIV-infected cells, which have the HIV envelop, can be recognized by the macrophage cells, and the recognition and contact between these cells enable the intercellular transfer of cGAMP; after promoting the anti-virus effect (83), this is also accepted as a mechanism of how ready is the uninfected cell to propagate the immune responses. In this sense, the cGAS-STING–mediated immune response pathway may act not only by inhibiting viral replication but also by inhibiting the spread of bystander cells to exert anti-viral effects. In addition to the cGAS-synthesized cGAMP, extracellular cyclic di-nucleotides (eCDNs) can activate the innate immune response in a particularly interesting mechanism. eCDNs are taken up by the macrophage cells through Clathrin-dependent endocytosis; after enhancing the DNA binding activity of cGAS, eCDN binding with cGAS further promotes cGAS-STING complex formation, which boosts the cGAS-STING–dependent innate immune response (84).

As mentioned above, several mechanisms exist, directly or indirectly, to activate cGAS-STING–mediated immune response; the activation of this DNA sensing pathway generates cytokine and chemokines, which can change the cell’s internal environment and then help cells against the infection events. Infectious disease, for example, Malaria, drives a p38-mediated IL6 production in macrophages and further induces pro-inflammatory monocytes to influence the T-cell function (85). Accumulated evidence indicates that cGAS-STING–mediated internal environment change affects the polarization of macrophage cells and ensue to influence T-cell differentiation (86, 87). From this, cGAS-STING–mediated immune response pathway bridges the innate and adaptive immune response to protect the host from better executing their function. However, cGAS-STING–induced inflammation is a troublesome problem that cannot be ignored; it has a tight connection with autoimmune diseases, neurodegeneration disease, and metabolic disorders. Reports indicate that tissue damage can release the host DNA to cytosol that triggers the cGAS-STING pathway activation in macrophages, further promoting the type I IFN production, macrophages recruitment, differentiation, and further inflammation (88–92). Although the effect of cGAS-STING pathway during the inflammation process is strong, how it influences the monocyte and macrophage functions still needs further study.

Cell aging refers to the change process in which the cell proliferation and differentiation ability and physiological function gradually decline over time in the process of cells performing life activities. Senescent cells that stop their proliferation secrete proteases, inflammatory cytokines, and growth factors; the senescence-associated secretory phenotype (SASP) accelerates senescent cell growth arrest and propagates growth inhibition in a paracrine manner (93). In the absence of cGAS or STING, SASP and immune cell infiltration are defective, and the clearance of RasV12-expressing cells is impaired leading to tumor development (94). Therefore, the cGAS-STING pathway senses DNA damage, induces SASP, and prevents tumorigenesis by enhancing senescence or enhancing the immune cell–mediated clearance of abnormal cells (95–97). STING also binds directly to bacterially produced cyclic dinucleotides, including cyclic di-GMP, cyclic diAMP, and bacterial cGAMP, all of which have traditional 3′,5′-phosphodiester linkages. DNA sensing by the cGAS-STING pathway also activates receptor-interacting serine/threonine-protein kinase 3 (RIPK3) and causes necrosis of bone marrow–derived macrophages (98). This pathway requires signaling through type I IFN receptors and tumor necrosis factor receptors, revealing a synergy of these pathways in their ability to induce cell death.

Undoubtedly, considering the central immune response function of the cGAS-STING pathway, although it is still a long way to go, the cGAS-STING axis already became a promising therapeutic target for cancer or autoimmune disease treatment.

## DNA damage repair and immune response

As the carrier of biological genetic information, DNA is polymerized from four deoxyribonucleotides: A (adenine), G (guanine), C (cytosine), and T (thymine), by a 3′,5′-phosphodiester bond. It mediates the replication of biological genetic information and acts as a template for transcription to guide protein biosynthesis, which plays an extremely important role in the growth and development of organisms. Cell growth and development as well as the stable transmission of genetic information between cells are closely related to the integration of the cell genome (99). During the transmission of genetic information, various endogenous and/or exogenous factors can cause abnormal changes in the composition and structure of DNA, affecting the stability of the genome. DNA damage is an inevitable event that can happen at any time due to exogenous and endogenous stress. When damage happens, the cell will activate a specific repair pathway according to the damage type to make sure that the genetic information is integrated to the maximum extent. Several repair pathways exist, such as Homologous recombination (HR), NHEJ, Base excision repair (BER), Nucleotide excision repair (NER), and Mismatch repair (MMR). There are many beautiful reviews already clarifying those DNA damage repair pathways (100–104). So far, the intersection between DNA damage response and the immune response is not a new topic (105, 106). In general, DNA damage can cause nucleus DNA to accumulate in the cytoplasm, which can activate immune response by the cytosol DNA immune receptors (105–110). Tumors and cancer cells that have DNA damage repair defects have more cytoplasm DNA enrichment and formed small but large impact DNA-containing structure: micronuclei (111, 112). Cytoplasmic micronuclei, a small DNA-containing structure in cytosol, resulting from defective DNA replication, DNA damage repair, or mitosis error, is a biomarker of DNA damage and genome instability and is considered as the major elicitor to trigger the cGAS-STING–dependent autoimmune response.

DNA damage response can cause the activation of an inflammatory response that increases the production of type I IFN and pro-inflammatory cytokines that promote innate immunity. The DNA damage repair factor ATM/ATR/Chk1 is required for the upregulation of NKG2DL, an innate immune system ligand for the NKG2D receptor (113). In uninfected non-tumor cell lines derived from humans and mice, expression of NKG2D ligands can be induced by DNA damage in an ATM- or ATR-dependent manner. Furthermore, these repair-related protein factors induce PD-L1 expression following IR or oxidative damage in a transcription-dependent manner (114, 115). TREX1, an important component of DDR, is also associated with autoimmune responses, and it can participate in DNA repair regulation and clear cytoplasmic DNA that activates innate immunity. Mutations in TREX1 in patients with Aicardi–Goutières syndrome and systemic lupus erythematosus result in the loss of its exonuclease activity (116–118). Autoantibodies against poly(ADP-ribose) polymerase (PARP), KU, DNA-PKcs, WRN, and MRE11 were identified in the serum of patients with systemic autoimmune rheumatism (119). It is precisely because the tight connection of DNA damage repair and immune response provided the opportunity to identify the ideal target for immunological disease and for cancer treatment.

## Cross-talk between cGAS-mediated immune response and DNA damage response

Because cGAS binds to dsDNA in a length-dependent but the sequence-independent manner, both foreign and host origin DNA can activate the cGAS-STING pathway. Therefore, controlling the availability of host DNA is essential to prevent aberrant pathway activation and inhibit autoimmune disease. There are several elegant mechanisms that exist to timely remove the abnormal DNA, such as DNaseII, TREX, and several DNA binding factors (111, 120).

As mentioned before, the genomic instability induced by DNA damage can cause micronuclei formation due to the abnormal processed DNA. During the repair process, unprotected ssDNA is easily digested or cleaved by various nucleases, inhibiting HR by preventing DNA entry and the formation and/or breakdown of Holliday junctions. These abnormal HR intermediates can stimulate cellular degradation of some or all damaged chromosomes, resulting in the release of nuclear DNA into the cytoplasm, which subsequently activates the cGAS-STING pathway and downstream immune responses. While mitotic error during cell segregation also can cause micronuclei formation that contains nucleus DNA, due to the micronuclei envelope being fragile and easily rupturing, released DNA from micronuclei was considered as the major reason that can activate cGAS-STING–dependent immune response (40, 121). This was affirmed by the cGAS that colocalizes with markers of DNA damage such as phosphorylated histone H2AX (γH2AX) in micronuclei (122, 123). Beyond that, Exo1 overexcites the DNA strand due to the loss of MutL protein homolog 1 (MLH1) (MutLα subunit), resulting in increased ssDNA formation (124). Depletion of RPA, DNA fragmentation, and the production of abnormal DNA repair intermediates all can release nuclear DNA into the cytoplasm and activate the cGAS-STING pathway (125). MLH1-deficient tumor cells accumulate cytoplasmic DNA to activate the cGAS-STING pathway, which sensitizes MLH1-deficient tumors to immunotherapy by promoting initiation and infiltration of antitumor CD8+ T cells. Disrupting the DNA-sensing functions of cGAS or STING renders MLH1-deficient tumors resistant to immunotherapy, which confirmed the DNA damage-induced immune response (126).

**Figure 1:**
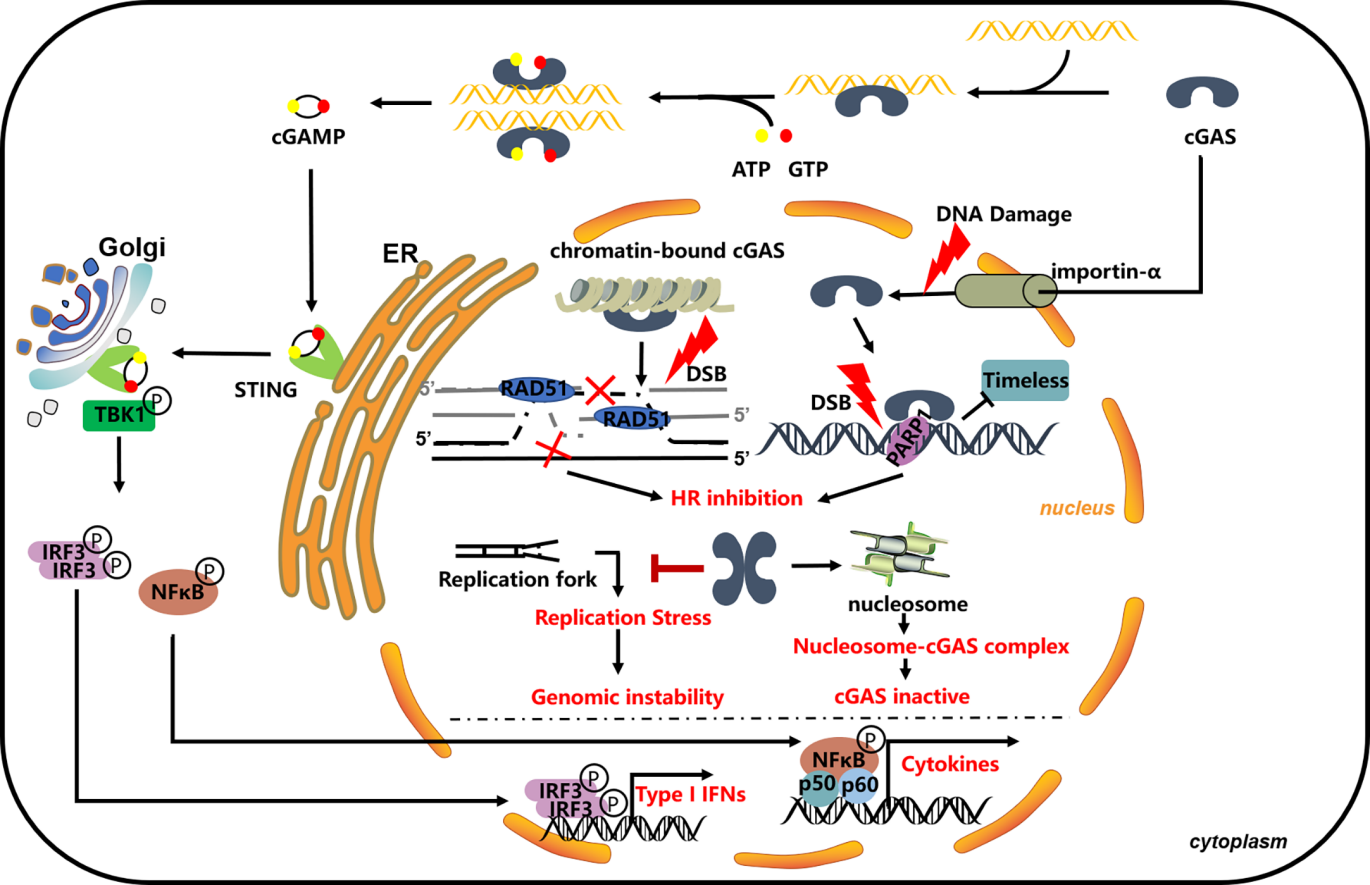
cGAS in DNA damage repair process. cGAS impaired the HR process through bind to chromatin and promoting its compaction, blocking the invasion of RAD51 and following the HR process (60); cGAS directly interacts with PARP1 and blocks its interaction with Timeless; the thought that it can be modified by PARP1 in the nucleus is unclear (63); cGAS can bind to the replication fork and slow down the replication progress and participate in replication stress-induced DNA repair (61).

In addition to the cytosol immune function of cGAS, new evidence indicates that the cGAS shutters between cytoplasm and nucleus. DNA damage induces cGAS to translocate into the nucleus or to export to cytosol under infection or through DNA damage treatment (127–130). In recent years, accumulated evidence indicates that cGAS is directly involved in the DNA damage repair process. Liu et al. (2018) first identified that cGAS participates in the DNA damage repair process; under different damage treatments, cGAS can transfer from the cytosol to the nucleus in a phosphorylation-dependent manner; translocated cGAS was recruited to the damage site by directly interacting with PARP1 in its enzyme activity-independent way. The interaction between cGAS and PARP1 impaired the connection between PARP1 and the DNA repair factor Timeless and then impaired PARP1-mediated HR repair (63). This work opens a new view of the function of cGAS in the nucleus. Subsequent work by Jiang et al. (2019) extended the function of cGAS in the DNA damage repair process; interestingly, they found that cGAS, as a chromatin-bound protein, is persistently located in the nucleus. This study confirmed the HR-involved function of cGAS, whereas the translocation of cGAS and its interaction with DNA repair factors may not the direct or critical part. cGAS is involved in the HR process in a phosphorylation-dependent manner, which is consistent with the study by Liu et al.; phosphorylation of cGAS also mediated its chromatin binding; meanwhile, this raises the possibility that Chromatin seems like a platform, mediating the concrete function of cGAS. In simple terms, the dimerization and chromatin-bound of cGAS are the key factors that direct cGAS DNA repair function: cGAS dimerization promotes chromatin compaction, which affects RAD51-mediated strand invasion step and eventually affects D-loop formation during the homology-mediated repair process and impaired the HR efficiency (60). These two different explanations exist to dissect the impact of cGAS on HR repair, and both works are valuable for researchers to understand the function of cGAS in the nucleus, although neither of them sheds light on how cGAS enzymatic activity was blocked in the nucleus. Several possibilities may be interesting to the researchers. As phase separation that may compact cGAS, nucleus DNA and related immune response proteins eventually prohibit the unwanted immune response; although cGAS recognizes DNA in a length-dependent sequence-independent manner, nucleus chromatin DNA is much more complex, such as chromatin bind protein effect, histone post-translation-modification, and the higher order of nucleus DNA; all these may change the patterns of nucleus DNA that is not preferred by cGAS again; post-translational modification (PTM) of cGAS is another very possible mechanism that can affect the cGAS function in the nucleus and then affect cGAS-mediated immune response function. In addition, even damaged DNA that is released from the DNA damage-trimming process can be recognized and bound by nucleus proteins, such as RPA and RAD51 (125), which also prohibit them to be recognized by cGAS. More than that, another study by Chen et al. (2020) further identified cGAS maintaining genome stability, particularly under replication stress: cGAS interacted with replication fork and regulated replication dynamics (61). Whereas, the function of cGAS in the replication process is still unclear; because cGAS constitutes binds and promotes chromatin compact, this may change the replication dynamic, especially under replication stress. Either way, cGAS is involved in the DNA damage repair pathway that is unquestionable (**Figure 1**). The discovery of cGAS in the nucleus and its function in the DNA damage response open a new area, triggering the researcher’s interest and posing some new interesting questions: Are there other mechanisms that exist to regulate the translocation of cGAS between cytoplasm and nucleus? Does translocation play a critical role in cGAS’s function under some circumstances—cGAS existing in cytosol and nucleus in the meantime? Is cGAS involved in other DNA damage repair pathways? Does cGAS activate the immune response in the nucleus, because cGAS was found as a chromatin-bound protein in the nucleus, and what mechanism restricts the recognition of cGAS to host genome DNA? All these questions need further investigation.

## Post-translational modification of cGAS in cytoplasm and nucleus

**Figure 2:**
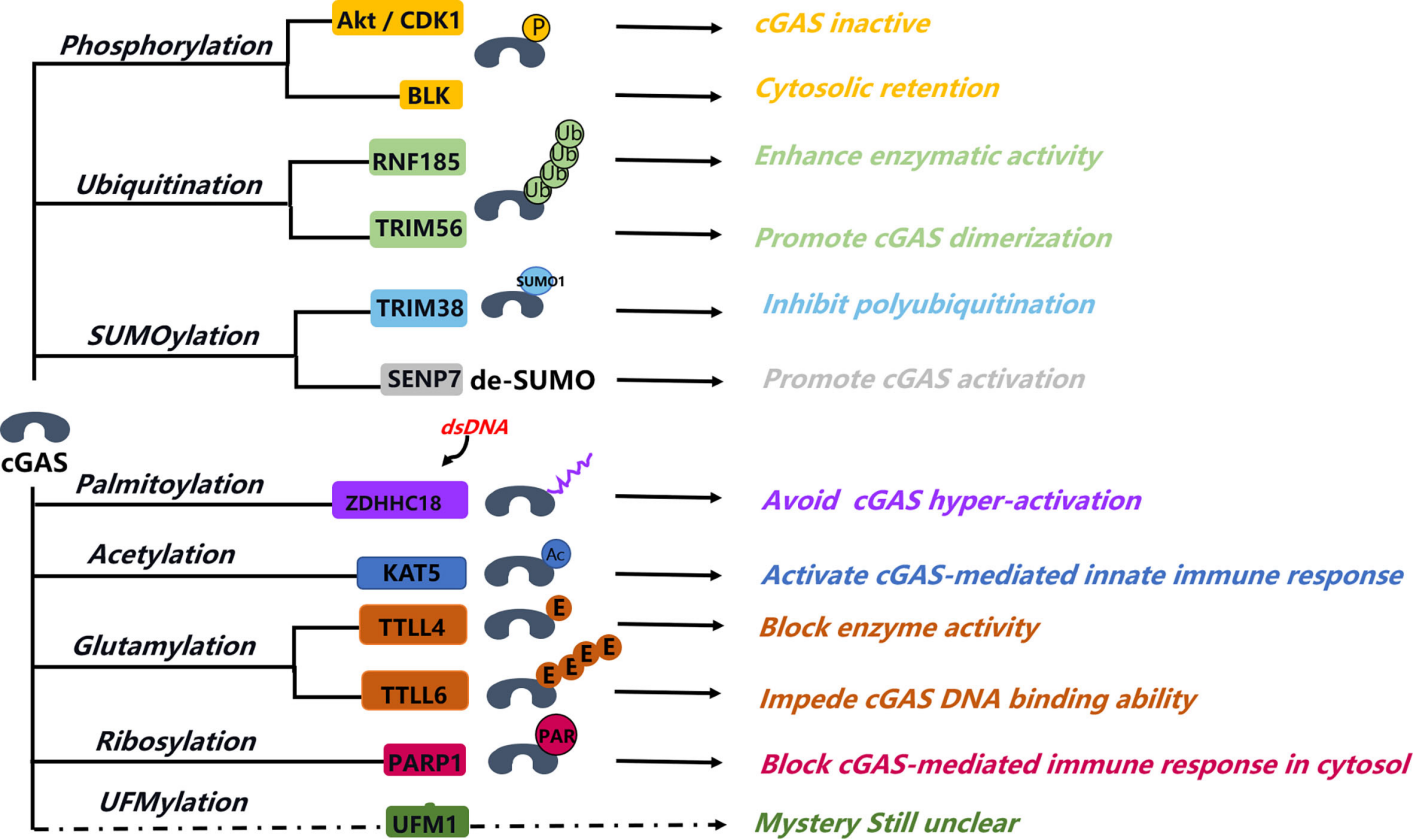
Post-translation modification of cGAS. Post-translation modification of cGAS involving in different aspects of the regulation of cGAS function, from activation to structure formation change, protein stability, DNA binding ability, and some unclear functions. Phosphorylation of cGAS is a major manner that can block cGAS activity in the nucleus; this help inhibits the abnormal autoimmunity response. Sumoylation of cGAS can coordinate with cGAS ubiquitination, fine-tuning its protein stability. Palmitoylation of cGAS blocks the interaction between cGAS and DNA and then affects the cGAS dimerization. Ribosylation of cGAS happened in the cytosol that can impede cGAS-mediated immune response. PARP1 interaction and mediated ribosylation of cGAS in cytosol that can attenuate its immune response activity, the interaction between PARP1 and cGAS in nucleus involved in the DNA repair process, and the function of ribosylation of cGAS in nucleus are unknown. UFMylation is a novel modification type, but its function in cGAS-STING–mediated immune response is still unclear.

As a pivotal immune response factor, cGAS is constitutively expressed in most tissues, although the fact that cGAS-STING-dependent immune response activation can feedback regulate the protein expression level of cGAS. The protein stability, enzymatic activity, DNA binding, and subcellular localization of cGAS are mainly affected by several different PTMs, such as phosphorylation, ubiquitination, PARylation, and SUMOylation (**Figure 2**). In the same way, PTMs may also be the major regulatory mechanism that can affect the activity and the related function of cGAS in the nucleus.

## Phosphorylation

Protein phosphorylation is the most basic, common, and reversible mechanism for regulating and controlling protein activity and function. The reversible cGAS phosphorylation mainly regulated its enzymatic activity from different layers. Akt kinase, for example, phosphorylation of mSer291 or hSer305 of cGAS, affects the cGAS activity under DNA virus stimulation (131, 132). The negative charge generated by phosphorylation of the N-terminal serine and threonine residues of cGAS is specific for its recognition of nuclear DNA and enables its activity to be selectively inhibited during mitosis (133). Phosphorylation of cGAS at S291/hSer305 by CDK1 during mitosis inhibits its activation by host genomic DNA, whereas protein phosphatase 1 (PP1) dephosphorylates this site after mitosis to restore the ability to sense cytoplasmic DNA (134). In addition to serine phosphorylation, cGAS can also be phosphorylated on its tyrosine residue, which is responsible for its nucleus localization and DNA repair function. In resting cells, cGAS relies on B lymphoid tyrosine kinases (BLKs) to phosphorylate the Y215 site to maintain its cytoplasmic localization, whereas cGAS nuclear translocation and its chromatin binding ability are dependent on its phosphorylation of Y215. DNA-damaging agents can dephosphorylate cGAS to initiate its nuclear translocation and recruitment to DSBs; cGAS nuclear localization, or its chromatin binding ability, is important for its inhibition of HR, as BLK knockdown and Y215E mutant result in increased cGAS nuclear translocation, eliminating cGAS suppression effect on HR (60, 127). Furthermore, cGAS is constitutively associated with the PP6 catalytic subunit (PPP6C) in resting cells, and dissociation of PPP6C occurs upon virus infection (135). The S420 site in the catalytic pocket of cGAS is phosphorylated due to the dissociation of PPP6C, which binds GTP and generates cGAMP (135), indicating that the phosphorylation level of the S420 site regulates the GTP binding ability. Because PPP6C was reported to be involved in the HR repair process and is also constitutively associated with cGAS (135, 136), the balance of cGAS’s phosphorylation regulated by PPP6C may affect its DNA damage repair efficiency in the nucleus. Overall, the reversible phosphorylation of cGAS is the major breakpoint that uncovers how cGAS activity restricts in the nucleus, although how the spatiotemporal regulation of this event is still under investigation.

## Ubiquitination

Ubiquitin modification is a cascade of enzymatic reactions regulated by a variety of enzymes. Similar to methylation, different from acetylation and phosphorylation, ubiquitination can act as inhibitory or activating depending on the specific ubiquitin type and modification site. Targeted protein can either be polyubiquitinated or monoubiquitinated. Ubiquitin can form variously linked polyubiquitin chains through its C-terminal binding to one of the seven lysines (K6, K11, K27, K29, K33, K48, and K63) on another ubiquitin. Dependent on the ubiquitination type, the ubiquitin modification regulates protein stability, localization, metabolism, activity, and so on (137–140). Up to now, several E3 ligases have been reported to regulate the activity and stability of cGAS, such as RNF185, TRIM56, and TIIM14. Most of these modifications focus on the cytosol function of cGAS; whether nucleus cGAS has specific ubiquitination modification has not been studied yet. RNF185 (ring finger protein 185) is an E3 ubiquitination ligase localized at the endoplasmic reticulum, which can interact with cGAS during HSV-1 infection. RNF185 mediates the formation of K27-linked ubiquitin chains of cGAS (K173 and K384 sites) and enhances its enzymatic activity; knockdown of RNF185 reduces the enzymatic activity of cGAS and inhibits the downstream IFN response (141). The E3 ubiquitination ligase TRIM56 was initially thought to be able to ubiquitinate STING protein and to activate the STING pathway. Whereas the in-depth study found that knockdown TRIM56 did not affect the direct activation of STING by cGAMP but rather enhanced the cGAS-STING pathway by mediating the ubiquitination of the K335 site of cGAS, promoting cGAS dimerization and cGAMP production (142). In resting cells, K48-linked ubiquitination occurs at the K414 site of cGAS, and p62 recognizes ubiquitinated cGAS and promotes its autophagic degradation, thereby inhibiting the type I IFN signaling pathway (143). When infected with DNA viruses, IFN-I induces the expression of TRIM14 and enforces the de-ubiquitination enzyme USP14 to degrade the cGAS ubiquitin chain connection at the K414 site, thus inhibiting the p62-mediated autophagy degradation of cGAS (143). Because ubiquitin modification can regulate protein function from different angles, including the protein shuttling between cytosol and nucleus (144–146), it will be interesting to identify ubiquitin modification of cGAS in the nucleus, which may help the researchers to better understand the shuttle mechanism of cGAS between cytosol and nucleus. In addition, these may give some clues on how cGAS can be regulated in the nucleus to restrict its activation to bind host nucleus DNA or regulate its DNA damage repair functions.

## SUMOylation

Many proteins share sequence homology with ubiquitin, and these ubiquitin-like proteins are divided into two categories: one is ubiquitin-related analogs with modification functions similar to ubiquitination, such as Rubl (Nedd8), Apg8, Apg12, and small ubiquitin-related modifiers (SUMO); the other is ubiquitin-domain proteins. Like ubiquitination, SUMOylation also results in the formation of an isopeptide bond between the glycine residue at the carboxyl-terminal of the modified protein and the ε-amino group of the substrate protein lysine. The specific pathway of Sumo modification is very similar to that of ubiquitination modification, involving a cascade reaction of multiple enzymes: E1 activating enzyme, E2 conjugating enzyme, and E3 ligase. However, the enzymes involved in the two reaction pathways are completely different. The SUMOylation modification process includes activation, binding, linking, modification, and other processes (147–149). In uninfected cells or during the early stages of viral infection, the ubiquitin ligase TRIM38 targets cGAS to SUMOylate and activates cGAS; in the late stage of infection, SENP2 mediates the deSUMOylation of cGAS to prevent its overactivation (150). SUMO linkages on lysine residues at positions 335, 372, and 382 of cGAS can significantly inhibit the binding of cGAS to DNA and reduce its oligomerization and nucleotidyl transferase activities. Meanwhile, SENP7-mediated deSUMOylation of cGAS can promote the activation of cGAS (151). However, as ubiquitination modification, all these studies suggested SUMOylation of cGAS is a cytosol event, either through regulating cGAS activity or stability, both can influence the cGAS-mediated immune response. Although SUMOylation in DNA damage repair is very common, many repair factors can be SUMOylate, such as MDC1, BRCA1, RPA, KU70, and PARP1 (152–159). SUMOylation of DNA repair factors can regulate their protein–protein interaction, degradation, localization, activity, and so on. Thus, this poses a possibility to investigate cGAS SUMOylation in the DNA repair process. Because cGAS SUMOylation can significantly inhibit its DNA binding activity and evidence indicates that cGAS is a chromatin-bound protein, SUMOylated cGAS is likely to regulate its DNA recognition function in the nucleus, thus affecting its DNA repair function or its activity to host origin DNA.

## Glutamylation

Glutamylation is an ATP-dependent process and is catalyzed/removed by glutamylases/carboxypeptidases (160, 161). Glutamylation was originally identified as a major modification of tubulins (162). These glutamylases include tubulin tyrosine ligase (TTL) and tubulin tyrosine ligase–like (TTLL) enzymes. TTLL6 can polyglutamylates cGAS at E272 sites, resulting in impeding its DNA-binding ability, whereas, at the E302 sites, mono-glutamylation of cGAS by TTLL4 blocks its enzyme activity (163). TTLL glutamylases seem to differ in their preferences for their substrates. Conversely, deglutamylation is hydrolyzed by cytosolic carboxypeptidases (CCPs). Among them, CCP1, CCP4, and CCP6 remove the shortening of penultimate polyglutamylation chains of α-tubulin; CCP5 removes the branch site glutamate. The study shows that CCP6 and CCP5 recover DNA binding and catalytic activity of cGAS by removing its polyglutamylation and mono-glutamylation, respectively (163). This finding suggested that dynamic glutamylation and deglutamylation of cGAS were tightly associated with its activity during HSV infection and shows a new regulatory mechanism of cGAS. However, further investigation into the mechanism of these enzyme activations still needs to be clarified.

## Acetylation

Protein lysine acetylation is involved in chromatin structure, metabolic functions, and transcriptional regulation (164). It is a reversible PTM controlled by lysine acetyltransferases (KATs) and lysine deacetylases (KADCs). Lysine acetylation was first found on histone acetylation; now, it has expanded from histone to lots of proteins in almost all cellular processes. Recently, cGAS activity was found to be regulated by acetylation. Dai et al. found that cGAS acetylation on one of the three lysine residues (K384, K394, and K414) contributes to its inactiveness (165). In response to DNA challenge, cGAS is deacetylated by HDAC3. It has also been suggested that lysine acetyltransferase 5 (KAT5) is a positive regulator of cGAS in response to DNA viral infection and dsDNA (166). KAT5 can acetylate cGAS at N-terminal K47, K52, K62, and K83 residues, increasing affinity to viral DNA and activating an innate immune response. Both studies helped to understand the delicate regulatory mechanisms of the innate immune response. Those C-terminal catalytic domains lysine residues (K384/394/414) and N-terminal unstructured domain lysine residues (K52/62/83) can be acetylated by two different regulatory mechanisms and cause different consequences, demonstrating the complexity of the same PTM on the same protein.

## Ribosylation

ADP-ribosylation is a critical reversible protein post-translation modification. Target proteins can be modified by single or multiple ribose and then can be involved in various cellular processes, including DNA damage repair and immune response. PARPi, which can block PARP1 activity or cause PARP1 trapping, is well known for its clinical treatment of HR deficiency disease. Evidence indicates that the PARPi can induce cytosol micronuclei formation due to the PARP trapping induced DNA damage and then trigger cGAS-STING–dependent immune response (167, 168). Although the nucleus function of cGAS in the DNA damage repair process is dependent on its interaction with PARP1, phosphorylated cGAS is translocated to the nucleus and interacted with PARP1 through PARP1-ADP-ribose, where cGAS can affect the HR repair through regulating the PARP1–TIMELESS interaction (63). Recently, there is another intriguing report by the same group indicating that DNA-pK can phosphorylate PARP1 and then further promote the export of PARP1 to the cytosol, PARylating cGAS in the cytosol that can block the cGAS-mediated immune response (169). This is a kind of feedback loop regulation mechanism because the nucleus PARP1 can PARylate DNA-pK to promote its activity. The connection between PARylation and cGAS became an interesting topic, and this opens another question of how cGAS activity inhibition in the nucleus as cGAS can be PARylated by PARP1 in the nucleus. It would be interesting to evaluate the function of cGAS PARylation in the nucleus, or whether it can restrict the function of cGAS to recognize the host DNA. This is highly possible because PARPi treatment that blocks PARP1 activity or induces PARP1 trapping promotes cGAS-STING–dependent immune response. Whether PARP1 activity during this process has contributed or not is still unclear, although cGAS PARylation in the cytosol by PARP1 indeed blocks the activity of cGAS to then attenuating cGAS-dependent immune response. Nevertheless, it is still possible that cGAS can be PARylated by PARP1 in the nucleus and thereby restricted cGAS reorganization of host DNA either by restricting its activity or blocking its interaction with host DNA.

## Protein arginine methylation

Protein arginine methylation is an important PTM in regulating multiple intracellular signaling, RNA processing, chromatin remodeling, and homologous recombination–mediated DNA repair. It is known to be catalyzed by protein arginine methyltransferase (PRMTs) in mammals; PRMTs catalyze the transfer of a methyl group from S-adenosylmethionine to the arginine residues of histone or non-histone proteins (170). PRMTs are categorized into three types based on the final methylarginine product that is generated: type I PRMTs (PRMT1, PRMT2, PRMT3, PRMT4/CARM1, PRMT6, and PRMT8), which catalyze the addition of a second methyl group to the nitrogen, producing asymmetric di-methylarginine (ADMA); type II PRMTs (PRMT5 and PRMT9), which methylate additional guanidine nitrogen, generating symmetrical demethylated arginine (SDMA); and type III PRMT (PRMT7), which generates monomethylated arginine (MMA).

Protein arginine methylation can influence the interaction between protein and DNA, as cGAS is the well-recognized cytosolic DNA sensor, thus indicating that protein arginine methylation may have a potential function in regulating cGAS. Ma et al. showed that PRMT5 is directly bound with cGAS and induced its asymmetric demethylation, which significantly attenuated cGAS-mediated anti-viral immune response (171). The study identified R124 residue as the direct catalyzing site by PRMT5; cGAS(R124K) mutant abolished PRMT5-mediated suppression of type I IFN production during virus infection. Furthermore, the R124 residue is a much-conserved motif in different species, indicating its significant importance for the anti-viral immune response mediated by cGAS. However, the protein demethylation mechanism responsible for fine-tuned modulation of cGAS anti-viral immune response is not known.

## Palmitoylation

Palmitoylation is an important PTM catalyzed by aspartate-histidine-histidine-cysteine (DHHC)-palmitoyl transferases, which participate in the regulation of diverse biological processes (172, 173). Hundreds of mammalian proteins have been discovered to be palmitoylated (174, 175). These proteins are either mono-palmitoylated or spontaneous autopalmitoylated; most of these proteins’ palmitoylation are executed by zinc finger aspartate-histidine-histidine-cysteine (DHCC)-type containing (ZDHHC) family of palmitoyl S-acyltransferases (PATs) (176). PATs are localized to the ER or Golgi apparatus, where STING transfers place under immune response activation. These linked the function of Palmitoylation with the cGAS-STING immune response pathway; indeed, growing evidence suggested in the cGAS-STING signaling pathway can be regulated by palmitoylation (177). Palmitoylation usually links fatty acids with the addition of a 16-carbon palmitic acid to cysteines *via* a thioester bond. It has been reported that activated STING translocated to the Golgi apparatus, where DHCC3, DHCC7, and DHCC15 palmitoylated at its Cys88 and Cys9 sites (177). Palmitoylated STING is essential for TBK recruitment and activation of the innate immune response.

Recently, Shi et al. suggested cGAS palmitoylation at Cys474 by ADHHC18, reducing its interaction with dsDNA (178). This study proposed an elaborate regulatory mechanism of cGAS: In the resting state in which cGAS is not palmitoylated, cGAS activates rapidly to initiate innate immune responses; upon cGAS recognizing and binding DNA, ZDHHC18 interacts and palmitoylates cGAS, leading to inhibiting cGAS-mediated innate immune signaling transduction. This identification of cGAS as a palmitoylated protein expands the knowledge of the role of palmitoylation in regulating protein function, which might provide new targets for drug development against viral infections and autoimmune diseases. However, palmitoylation that controls the cGAS-STING function remains to be fully defined.

## UFMylation

UFMylation is a ubiquitin-like modification catalyzed by ubiquitin-like modification activating enzyme 5 (UBA5), ubiquitin fold modification conjugating enzyme 1 (UFC1), and UFM1-specific ligase 1 (UFL1) (179). In general, UFM1 is an inactive precursor form (Pro-UFM1) that elicits a continuous binding reaction when its C-terminal Gly residue is exposed by UFM1-specific proteases (UFSPs). Then, the only E1 enzyme UBA5 activates UFM1 with the help of ATP in the cytoplasm. Similar to other E2 responses, activated UFM1 is transferred to the UFM1 E2-conjugating enzyme UFC1. Finally, E3 ligase helps transfer UFM1 from UFC1 to the substrate. UFSPs can also cleave UFM1 from its target protein, making UFM modification reversible (180, 181). UFMylation has a variety of important biological functions and participates in various biological regulatory processes such as endoplasmic reticulum stress, embryonic development, and DNA damage repair (182–186). UFMylation plays a role in the DNA damage response and regulates ataxia-telangiectasia–mutated (ATM) signaling in response to genotoxic agents (187, 188). DNA damage and ATM loss of function have been shown to activate type I IFN signaling and to amplify cGAS-STING–dependent innate immune responses (189, 190). Ufmylation pathway can be a potential critical modification manner that is involved in immune response; in addition, a new report indicates that abrogating any factors of the UFM pathway can induce the upregulation of the expression of innate and adaptive immune response factors (191). Hence, understanding whether the UFM system is involved in the cGAS-STING pathway is developing research. UFMylation plays an important role in ER, whereas the cGAS-STING function centers in the cytosol. Given that UFMylation has a critical function in DDR, we can see that it may have a great potential in regulating cGAS in the nucleus.

## Conclusion marks

Many conventional cancer treatments are based on that tumor cells are extremely sensitive to DNA damage. Radiotherapy and chemotherapy are used to cause DNA damage in tumor cells to achieve therapeutic effects. In addition to causing cell cycle arrest and cell death, DNA damage can activate the immune system. The immune responses that are damaged by chemotherapy and radiotherapy are important for the efficacy of cancer therapy and are mediated, at least in part, by the tumor cell’s intracellular responses. Although studies have shown that damaged cells can secrete type I IFNs and pro-inflammatory factors, the current research on the molecular mechanisms between DNA damage and innate immune signaling is only the tip of the iceberg and needs to be resolved deeply. Recent studies have shown that cGAS exists in the nucleus and is associated with DNA damage-induced genomic instability. DNA damage itself leads to the formation of micronuclei; cGAS in the cytoplasm can colocalize with and recognize broken DNA in micronuclei, following the activation of the cGAS-STING–dependent immune response. cGAS-dependent genome instability in the nucleus and genome instability related to the immune response in cytosol further make it critical to understand the shuttle mechanism of cGAS between cytoplasm and nucleus, and how cGAS was restricted to recognize self-DNA in unperturbed conditions.

Recently, several elegant reports try to uncover the nucleus cGAS inhibition mechanism from the structural standpoint, from their results, cGAS bound to the nucleosome, attached to the nucleosome surface “acidic patch”, the H2A-H2B interface area that serves as a platform to regulate the protein–chromatin interaction. These studies revealed how nuclear cGAS is inhibited by the histone H2A-H2B and nearby nucleosomes (192–197); however, they ignored the effect of the post-translation modification, coming from either histones or cGAS. In addition, because cGAS dimerization promotes chromatin compaction, this may change the three-dimensional structure of chromatin, which makes the situation more complex. RNA binding is another very interesting discovery that can regulate cGAS activity in the nucleus; in this study, the circular RNA cia-cGAS was discovered to bind cGAS tightly and block its activity, following cGAS-mediated immune response (198). Although the circular RNA–mediated cGAS inhibition is a universal mechanism or just tissue, cell type specific still need further investigated.

CDK-mediated phosphorylation of cGAS inhibits its activity in the nucleus, especially in the mitotic stage where DNA is unwrapped and not protected by histones. cGAS is recruited to DNA damage sites to regulate HR repair, but how cGAS affects the HR repair efficiency is still a mystery. Damaged DNA can be a potential substrate for cGAS recognition which may trigger the sterile inflammation response. cGAS directly interact with PARP1and PARylation of cGAS in cytoplasm attenuate the immune activity; therefore, cGAS may be a substrate of PARP1 in the nucleus that also blocks its activity. Meanwhile, DNA-pk phosphorylation of PARP1 promotes the translocation of PARP1 from the nucleus to cytosol, raising the possibility that post-translation modifications of cGAS can influence its shuttle between the nucleus and cytosol.

UFMylation modifications are currently reported to have an important role in participating in the DNA damage response; the key UFMylation enzyme, UFL1, regulates the immune response by affecting the protein stability of STING. However, the regulation of cGAS through UFMylation modifications is unclear; if so, the function of cGAS UFMylation in the DNA damage repair process and immune response pathway needs to be further investigated. Exploring the relationship between UFMylation modification, cGAS-dependent immune response signaling, and DNA damage repair pathway may provide new targets for cancer prevention and treatment.

Up to now, most of the studies about cGAS-STING focus on its cytosol immune response function. Because more and more evidence approved that cGAS is permanently localized in the nucleus as a chromatin bind protein, it will be interesting to investigate its nucleus function, especially from the post-translation modification standpoint; whether these PTMs of cGAS, such as ubiquitination, SUMOylation, methylation, and acylation, happened in the nucleus is an interesting and urgent question. As most of these PTMs were reported to regulate the activity of cGAS, do they play the major roles to restrict the function of cGAS in nucleus or not? If not, what are the underlying mechanisms to separate the cytosol and nucleus cGAS? Further question is how these PTMs of cGAS coordinated with each other to fine-tuning the cGAS function, from its activity, protein–protein interaction, chromatin binding, chromatin release, nucleus to cytosol shutting to sterling immune inhibition, and so on.

## Data availability statement

The original contributions presented in the study are included in the article/supplementary material. Further inquiries can be directed to the corresponding author.

## Author contributions

J-XS and MZ wrote the manuscript with input from all authors. All authors contributed to the article and approved the submitted version.
